# Clinical differences between periprosthetic and native distal femur fractures: a comparative observational study

**DOI:** 10.1186/s13018-024-04796-8

**Published:** 2024-05-20

**Authors:** Shana Kong, Shannon Tse, Aziz Saade, Barry Bautista, Max Haffner, Augustine M. Saiz

**Affiliations:** Department of Orthopaedic Surgery, University of California Davis, 4860 Y Street, Suite 3800, Sacramento, CA 95817 USA

## Abstract

**Introduction:**

The incidence of periprosthetic distal femur fractures (PDFF) is increasing as the number of total knee replacements becomes more common. This study compared the demographics, fracture characteristics, treatment, and outcomes of periprosthetic versus native distal femur fractures (NDFF).

**Materials and methods:**

This was a retrospective cohort study of patients ≥ 18 who underwent surgical fixation of NDFF or PDFF from 2012 to 2020 at a level-1 trauma center. The main variables collected included demographics, AO/OTA fracture classification, fixation construct, concomitant fractures, polytrauma rates, bone density, and reduction quality. Primary outcomes were unexpected return to the operating room (UROR), hospital length of stay, and quality of reduction. T-tests, Fisher’s exact tests, and multivariate analyses were used for statistical analysis.

**Results:**

209 patients were identified, including 70 PDFF and 139 NDFF. PDFF patients were elderly females (81%) with isolated (80%) and comminuted (85%) 33 A.3 (71%) fractures. NDFF patients included 53% females, were commonly middle-aged, and displayed comminuted (92%) 33 C.2 fractures. 48% of NDFF patients had concomitant fractures. Intramedullary nailing was the primary fixation for both groups, followed by nail-plate combination (37%) for PDFF and lateral locking plates (21%) for NDFF. NDFF patients experienced significantly longer hospital stays, higher UROR rates, and worse quality of reduction (*p* < 0.05). PDFF patients had a significantly greater prevalence of low bone density (*p* < 0.05).

**Conclusion:**

PDFF occur as isolated injuries with significant metaphyseal comminution in elderly females with low bone quality. NDFF commonly occurs in younger patients with less metaphyseal comminution and concomitant fractures. Intramedullary nailing was the most common treatment for both groups, although preference for nail-plate combination fixation is increasing. NDFF type 33 C fractures are at greater risk of UROR.

## Introduction

In 2012, total knee arthroplasty (TKA) was the single most common surgical procedure performed in the United States, with 94% of those procedures occurring in patients 65–84 years old [1, 2]. As the population ages and the prevalence of TKA rises, the frequency of periprosthetic distal femur fractures (PDFF) has simultaneously increased. Multiple studies report that periprosthetic distal femur fractures are not uncommon following TKA [3–5]. Court-Brown et al. reported an increase in PDFF prevalence from 15.4 to 27.8% of all distal femur fractures from 2007 to 2011 [6]. Treatment of PDFF presents a challenge for orthopedic surgeons, who must consider not only the complex and variable fracture morphology associated with the prosthesis but also the specific populations at risk.

Previous studies have focused on analyzing PDFF and native distal femur fractures (NDFF) separately. Roy et al. analyzed NDFF exclusively at a single level-1 trauma center and characterized the affected population as middle-aged, female (66%), and more often overweight than osteoporotic [7]. Additionally, they identified similar rates of high-and low-energy trauma in their NDFF cohort and found that high-energy injuries were associated with more severe fracture types (AO/OTA 33B/C), open fractures, and additional orthopedic injuries, while low-energy injuries were associated with closed fractures and less severe fracture types (AO/OTA 33 A) [7]. Elsoe et al. first reported on the demographics of PDFF, noting a population distribution of elderly females (mean age = 77) with low-energy injuries [8]. Regarding treatment, lateral plating and retrograde intramedullary nailing (rIMN) have been reported as the most common and successful fixation methods for both PDFF and NDFF, although intramedullary nail-plate combination (NPC) fixation is a more recent technique developed to allow quicker weight bearing and achieve better purchase in osteoporotic bone [7, 9–12]. Studies have reported similar 30-day and 90-day outcomes [13, 14]. A previous case series reported that osteoporosis, comminution, intra-articular involvement, and soft tissue injury in open fractures were associated with worse long-term (> 5 years) outcomes such as malunion, nonunion, and infection in NDFF [15]. Reduction quality has yet to be studied in either group.

In the existing body of literature, there is a notable lack of comprehensive comparisons between PDFF and NDFF within single institutions. Studies have focused exclusively on one fracture type without directly comparing it with the other. As a result, there is limited understanding of the relative differences between PDFF and NDFF in terms of demographics, fracture characteristics, treatment modalities, and outcomes, as patients from both groups can only be compared from different studies, which potentially raises concerns such as selection bias, confounding, heterogeneity, and timeframe variations with historical comparisons.

While there is some consistency in the literature regarding these fracture types, there remains a need to systematically examine and compare them within the same population to further denote the remaining discrepancies which may arise from limited generalizability. Therefore, the primary objective of this study was to conduct a detailed comparison of PDFF and NDFF using data from a single institution. This paper contributes to our understanding of the differences in patient populations, specific injury patterns, and treatment strategies between periprosthetic and native distal femur fractures. Such insights are valuable for patient counseling. Moreover, attempting to adapt fixation strategies from one to the other may prove ineffective, thus prompting avenues for further exploration.

A secondary aim was to explore aspects such as the quality of reduction and long-term outcomes, which have not been extensively studied in prior literature. NDFF often have more complex fracture morphology and are not only more difficult to reduce but are further complicated by the need to restore length, planar alignment, and joint line congruity. In contrast, PDFF tend to be simpler fractures, but it can be challenging to achieve anchorage and maintain adequate fixation in highly comminuted, osteoporotic bone [16]. Studying reduction quality and long-term outcomes in these groups would provide valuable insight on whether current treatment strategies are achieving optimal fixation for both groups, and could guide future, more personalized treatment strategies to improve patient outcomes.

We hypothesize that PDFF are more likely to be isolated, simple, low-energy, injuries experienced by elderly patients while NDFF will be experienced by a younger population and result in more complicated fracture patterns due to higher levels of trauma.

Furthermore, we hypothesize that there will be no difference in postoperative outcomes between PDFF and NDFF groups.

## Methods

### Cohort selection

This retrospective study was approved by the Institutional Review Board. All patients who were at least 18 years of age at the time of admission with distal femur fractures between January 2012 and December 2020 at a single level-1 trauma center were identified. These patients were later classified as either NDFF or PDFF. Patients with bilateral distal femur fractures were excluded.

### Data collection

**Fig. 1:**
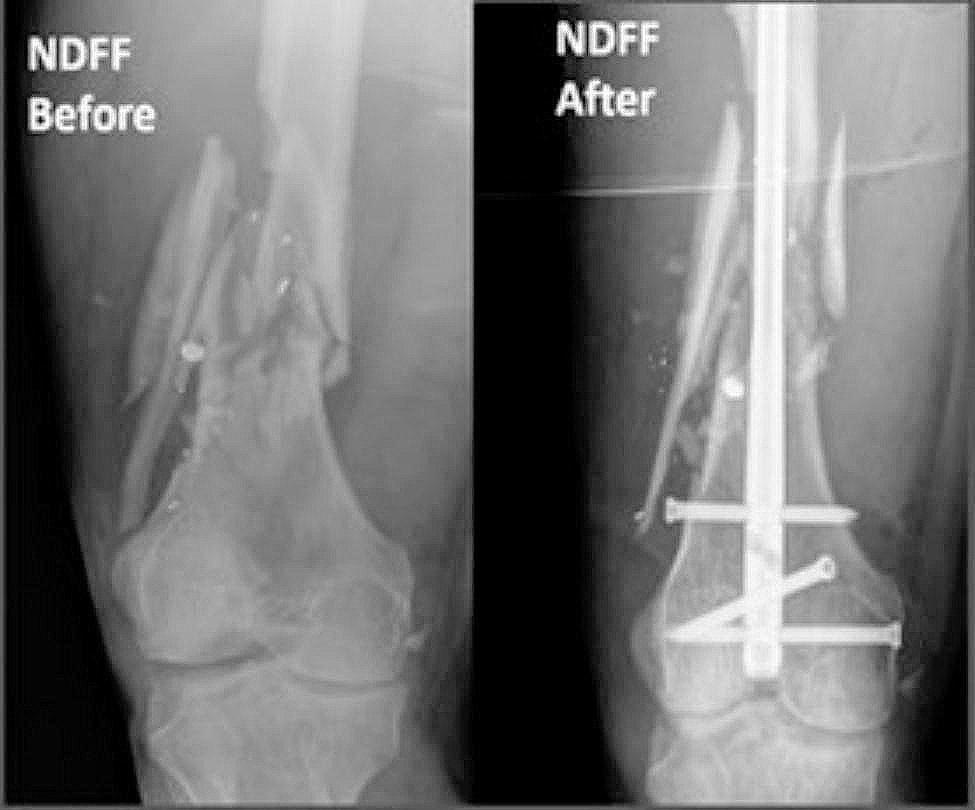
Preoperative (left) and post-IMN (right) NDFF radiographs

**Fig. 2:**
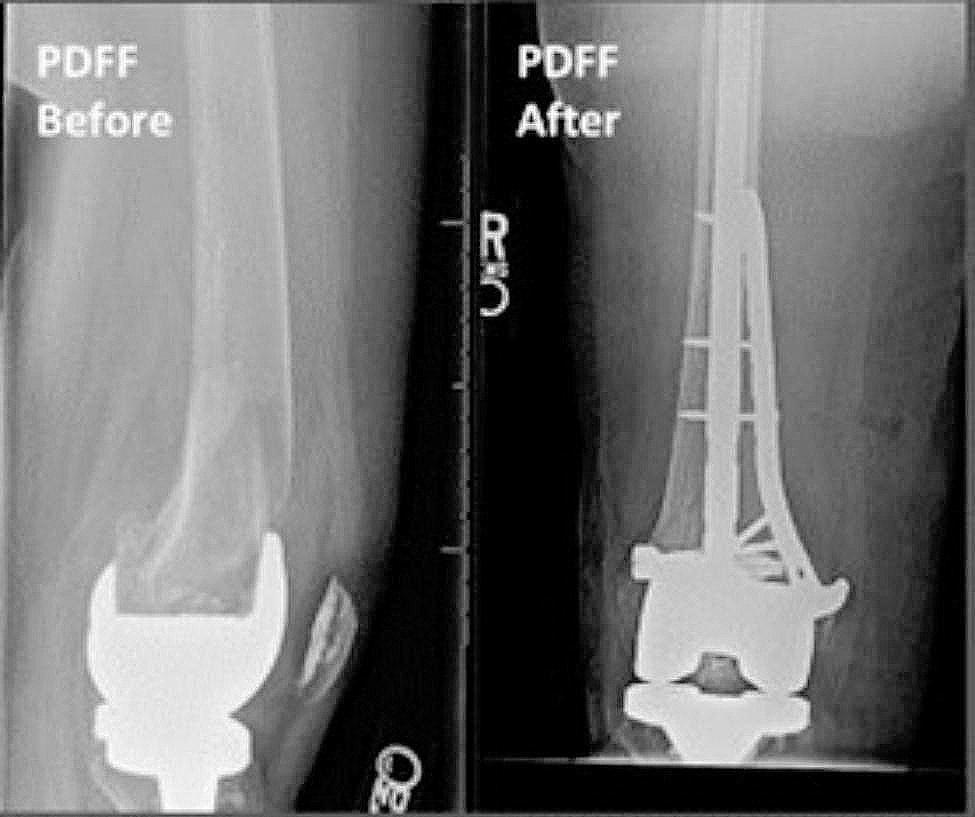
Preoperative (left) and post-NPC (right) PDFF radiographs

Electronic medical records were used to obtain clinical and demographic data for each patient. American Society of Anesthesiologists (ASA) score and bone density status were obtained from clinical records. Presence of bone loss, classified as either osteopenia or osteoporosis, was determined by either previous diagnosis documented in the patient chart with a prior DEXA scan or by comments noted by radiologists from existing radiographs following a fragility fracture. Fracture characteristics, including AO/OTA fracture classification, were obtained from preoperative radiographs (Figs. 1 and 2) and computed tomography (CT) scans. Polytrauma was noted if present and defined as having two or more injuries affecting two or more bodily areas. Type of surgical fixation was obtained from operative notes and postoperative radiographs (Figs. 1 and 2). Patients with NPC fixation were categorized separately and did not contribute to nail or plate counts. Primary outcomes were unexpected return to the operating room (UROR), hospital length of stay, and quality of reduction. Postoperative outcome data was obtained using follow-up notes and postoperative radiographs.

**Fig. 3:**
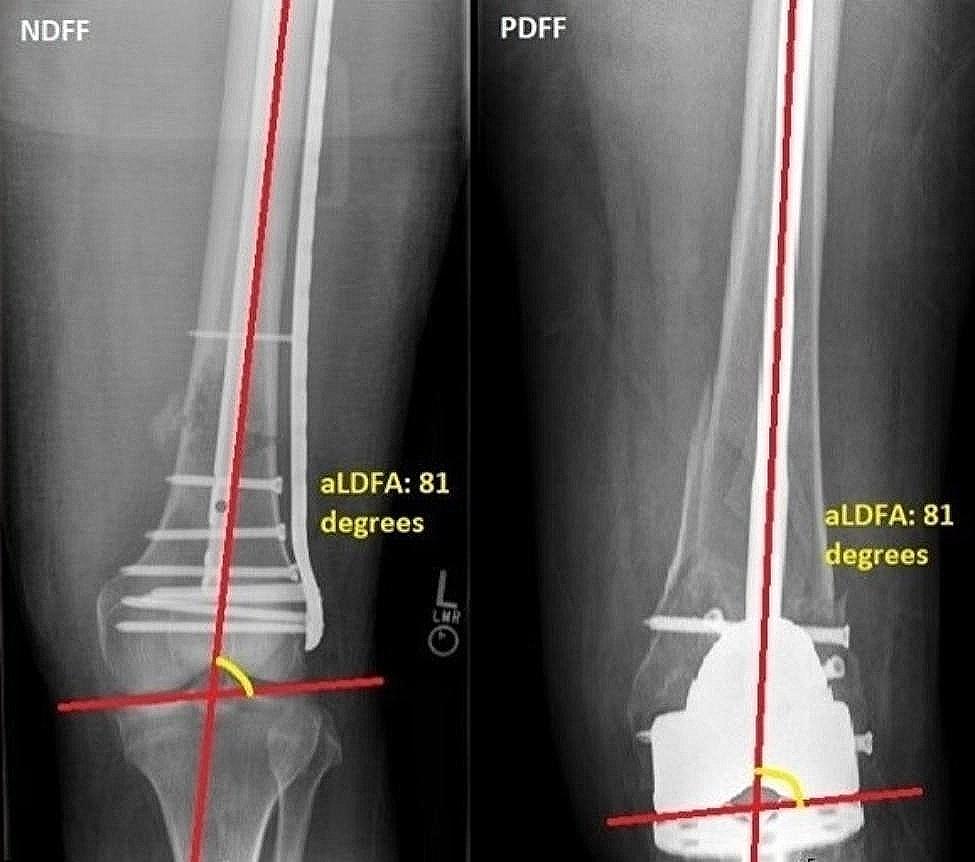
Measurement of aLDFA on AP radiographs

Quality of reduction was assessed by calculating the difference in alignment from the population average anatomic lateral distal femoral angle (aLDFA) on postoperative anteroposterior (AP) radiographs. These measurements were made by a single trained grader. To measure the aLDFA (Fig. 3), a line was first drawn parallel to both femoral condyles of the affected femur, representing the knee joint line. Next, a line was drawn from the center of the femoral head to the intercondylar notch of the affected femur, representing the mechanical axis of the femur. The lateral angle formed between the mechanical axis of the femur and the knee joint line was recorded in degrees as the aLDFA. The aLDFA for the contralateral, unaffected femur was also calculated for use as a reference for each patient if contralateral films had been acquired. The mean difference from the published population average aLDFA of 81 degrees was calculated in the affected and unaffected femurs for both PDFF and NDFF groups [16–19]. A smaller deviation from the population average aLDFA was accepted as a more desirable outcome.

### Statistical analyses

Differences between PDFF and NDFF patients were compared using Fisher’s exact tests, unpaired t-tests, or multivariate analyses, as appropriate for each variable using Microsoft Excel. Multivariate analysis was completed in Excel using a linear regression with all variables included. Results were described using incidence rates, means, and one standard deviation. A p-value < 0.05 indicated statistical significance.

## Results

In total, 209 patients were included in this study. Table 1 summarizes the preoperative characteristics of both cohorts. The mean age of NDFF patients was 23 years younger than the PDFF patients (*p* < 0.001). There were significantly fewer females in the NDFF cohort (53% compared to 81%, *p* < 0.001). The PDFF cohort had significantly higher rates of bone loss (55.7% compared to 19.4%, *p* < 0.001). There was no significant difference between the ASA scores of the two cohorts (*p* = 0.061).

**Table 1:** Preoperative characteristics of NDFF and PDFF patients

Measure	NDFF	PDFF	*P*-value
Number of Cases	139	70	
Age (years)	57 ± 19.7	80 ± 10.1	**< 0.001** ^**a**^
Gender (female rate)	53%	81%	**< 0.001** ^**b**^
Presence of bone loss (Osteopenia or Osteoporosis)	19.4%	55.7%	**< 0.001** ^**b**^
ASA Status: class 1–2, 3–4	32.4%, 67.6%	20%, 80%	0.061^b^

^a^ t-test

^b^ Fisher exact test

Table 2 summarizes the fracture characteristics of the two cohorts. 33 A.2 and 33 A.3 classifications represented the entirety of PDFF. In comparison, NDFF AO/OTA classifications were represented by 33 C.2 (28%), 33 C.3 (22%), and 33B.1 (12%), which were significantly more prevalent than in the PDFF cohort (*p* < 0.001 for 33 C.2 and 33 C.3, and *p* < 0.05 for 33B.1).

**Table 2:** Fracture characteristics of NDFF and PDFF patients

	NDFF		PDFF	*P*-value
AO/OTA classification				
33 A.2 Extra-articular Simple fracture	6%		29%	**< 0.001** ^**a**^
33 A.3 Extra-articular Wedge or Multifragmentary Fracture	25%		71%	**< 0.001** ^**a**^
33 C.1 Simple Articular, Simple metaphyseal	6%		0%	0.215^a^
33 C.2 Simple Articular Wedge or Multifragmentary Metaphyseal	28%		0%	**< 0.001** ^**a**^
33 C.3 Multifragmentary Articular, Simple, Wedge or Multifragmentary metaphyseal	22%		0%	**< 0.001** ^**a**^
33B.1 Partial Articular Lateral Condyle	12%		0%	**< 0.05** ^**a**^
33B.3 Frontal/Coronal	2%		0%	0.552^a^
Isolated Injury	51.8%		80%	**< 0.001** ^**a**^
Polytrauma	41%		18.6%	**< 0.001** ^**a**^
Comminution	85%		92.1%	0.274^a^
Interprosthetic Fracture	-		24.3%	-
Open	30.9%		11.2%	**0.002** ^**a**^
Gustilo-Anderson Type I	9%		25%	
Gustilo-Anderson Type II	14%		50%	
Gustilo-Anderson Type III	77%		25%	

^a^ Fisher’s exact test

Table 2 also shows significant differences in the rate of isolated injuries and polytraumas between both cohorts. PDFF were more often isolated injuries in patients (80% versus 51.8%, *p* < 0.001). NDFF patients were more likely to be polytraumatized (41% compared to 18.6%, *p* < 0.001). Additionally, NDFF patients had higher rates of open fractures (30.7% compared to 11.2%, *p* = 0.002). Out of 43 open NDFF fractures, 77% were Gustilo-Anderson type III open fractures (31 type 3 A, 2 type 3 C), 14% type II fractures, and 9% type I fractures (Table 2).

Of these open fractures, 41 underwent definitive fixation at first operation while 2 initially underwent external fixation due to the presence of an unstable knee (multiligamentous injury, fracture-dislocation). Out of 8 open PDFF fractures, 50% were Gustilo-Anderson type II, 25% type III, and 25% type I (Table 2). Only one PDFF patient was initially temporized with an external fixator before definitive fixation. There was no significant difference between the comminution rates of fractures in both cohorts (*p* = 0.274).

Table 3 summarizes the surgical fixation constructs used for both cohorts. Retrograde intermedullary nail was the most common fixation utilized in both groups, and there was no significant difference in the prevalence of rIMN between the two groups (NDFF = 36.7%, PDFF = 44.3%, *p* = 0.297). NDFF patients had significantly higher rates of medial plate fixation (5.8% compared to 0%, *p* < 0.05) and PDFF patients had significantly higher rates of NPC fixation (37.1% compared to 17.2%, *p* < 0.05). Further analysis of NPC fixation revealed usage beginning in 2015 for both groups, with prevalence of usage increasing from 3.3% and 0.7–13.3% and 4.3% in 2019 (PDFF) and 2020 (NDFF) respectively. Of 13 PDFF that received plate fixation, only 2 had fracture patterns amenable for nail fixation but preexisting implants (long-stemmed proximal component, Schneider rods for osteogenesis imperfecta) precluded intramedullary fixation. The 11 PDFF patients that received plate fixation had stable femoral components and fracture morphology that could not be effectively reduced with intramedullary fixation.

**Table 3:** Surgical fixation constructs used in NDFF and PDFF patients

Fixation construct	NDFF	PDFF	*P*-value
Intermedullary Nail	36.7%	44.3%	0.297^a^
Nail Plate Combination	17.2%	37.1%	**< 0.05** ^**a**^
Lateral Plate	20.8%	14.3%	0.266^a^
Medial Plate	5.8%	0%	0.053^a^
Lag Screws	6.5%	0%	**< 0.05** ^**a**^
Other	12.9%	4.3%	-

^a^ Fisher’s exact test

Postoperative outcomes are summarized in Table 4. Patients in the NDFF cohort had an approximately five-day longer length of stay compared to those in the PDFF cohort (*p* < 0.05). This was most likely because NDFF patients had more polytrauma (41% vs. 18.6%, *p* < 0.001) and open injuries (30.7% vs. 11.2%, *p* = 0.002) There was no significant difference in length of follow-up between the two cohorts (*p* = 0.564). Postoperatively, PDFF were significantly more likely to be weight bearing as tolerated (WBAT) compared to partial weight bearing (PWB) or non-weight bearing (NWB). NDFF postoperative weight bearing status consisted of 60% NWB, 21% WBAT, and 19% PWB. There was some correlation between postoperative weight bearing status and fixation. All NDFF patients who were WBAT underwent IMN, NPC, or lag screw fixation. Of patients who were NWB, 50% received plate fixation. There was no significant difference in WBAT assignment for NDFF who received NPC vs. nail or plate fixation alone (*p* = 0.88).

**Table 4:** Postoperative outcomes of PDFF and NDFF patients

Measure	NDFF		PDFF	*P*-value
Any Re-operation Occurrence	19 (13.6%)		6 (8.5%)	**< 0.05** ^**a**^
Malunion	1 (0.7%)		1 (1.4%)	--
Nonunion	4 (2.8%)		2 (2.8%)	--
Revision	1 (0.7%)		1 (2.9%)	--
Irrigation and Debridement	7 (5%)		0	--
Stiff Knee	2 (1.4%)		0	--
Painful Orthopedic Hardware	0		2 (2.8%)	--
Reinjury	1 (0.7%)		0	--
Septic Arthritis	1 (0.7%)		0	--
Skin Necrosis	1 (0.7)		0	
Foreign Body Removal	1 (0.7%)		0	--
Postoperative Weightbearing Status (%WBAT)	21%		60%	**< 0.001** ^**a**^
Length of Stay (days)	11.4 ± 16.6		6.36 ± 5.74	**< 0.05** ^**b**^
Length of Follow-up (days)	148 ± 181		164 ± 204	0.564^b^
Deviation of affected side from standard aLDFA (degrees)	9.06 ± 11.1		3.04 ± 2.86	**< 0.001** ^**b**^
Deviation of contralateral side from standard aLDFA (degrees)	2.92 ± 3.97 (*n* = 9)		1.7 ± 0 (*n* = 1)	^**−−**^
^a^ Fisher’s exact test				

^b^ t-test

For NDFF patients who were NWB or PWB, there was no correlation with fixation. However, there was a correlation between weight bearing status and polytrauma. Rates of polytrauma were 99% and 52% in patients who were NWB and PWB, respectively. Additionally, a majority of patients (60%) who received IMN fixation and were PWB had concomitant extremity fractures, preventing them from having increased weight bearing.

PDFF postoperative weight bearing status consisted of 60% WBAT, 30% PWB, and 10% NWB. Again, there was some correlation between postoperative weight bearing status and fixation. 100% of patients who were WBAT underwent IMN or NPC fixation, while 50% of patients who were TDWB underwent plate fixation. PDFF that received NPC fixation were significantly more likely to be assigned WBAT compared to those that underwent nail or plate fixation alone (*p* < 0.05). For patients who were PWB or NWB, there was no correlation between postoperative weight bearing status and fixation.

Compared to their PDFF counterparts, NDFF patients had higher rates of any unexpected return to the operating room (UROR) occurrences (13.6% compared to 8.5%, *p* < 0.05). Among the 19 NDFF requiring reoperation, 10 (52%) were AO/OTA type 33 C fractures. Irrigation and debridement (I&D) of the femur fracture of interest due to infection was the leading cause of UROR for NDFF patients (5% compared to 0%). NDFF UROR patients that initially experienced open fractures had a significantly greater risk of returning for I&D compared to closed NDFF fractures, likely due to an increased risk for infection (*p* < 0.05, Table 5). The most common reason for revision operation within the NDFF cohort was due to the intra-articular prominence of an intramedullary nail, and the other revision within the PDFF cohort was due to implant failure with valgus angulation. PDFF patients had significantly improved quality of reduction with respect to the average population aLDFA of 81 degrees. The mean aLDFA in PDFF patients was approximately 6 degrees less than that of NDFF patients (*p* < 0.001). When using the uninjured contralateral aLDFA, PDFF were approximately 1.2 degrees less in deviation. However, significance could not be determined given the limited availability of contralateral imaging.

**Table 5:** Postoperative outcomes of open versus closed NDFF and PDFF

Measure	Open NDFF		Closed NDFF	*P*-value	Open PDFF	Closed PDFF	*P*-value
Total	43		96	--	8	62	0.98 ^a^
Any Re-operation Occurrence	8		11	0.15^a^	0	6	
Malunion	0		1 (9%)	--	0	1	--
Nonunion	2 (25%)		2 (18%)	--	0	2	--
Revision	0		1 (9%)	--	0	1	--
Stiff Knee				--		0	
Irrigation and Debridement	5 (62.5%)		2 (18%)	*p* < 0.05^a^	0	0	--
Painful Orthopedic Hardware	0		0	--	0	2	--
Septic Arthritis	0		1 (9%)	--	0	0	--
Skin Necrosis	1 (12.5%)		0	--	0	0	

^a^ Fisher’s exact test

Of 26 PDFF treated with NPC fixation, only 1 (4%) experienced long-term post-operative complications of malunion. Of 44 PDFF treated with either nail or plate fixation, 2 (4.5%) experienced postoperative complications of nonunion. There was no significant difference in malunion/nonunion rates between these two groups (*p* = 0.88).

All 24 NDFF treated with NPC fixation achieved fracture union. Of 115 NDFF treated with either nail or plate fixation, 5 experienced malunion/nonunion. There was no significant difference in malunion/nonunion rates between NDFF treated with NPC fixation compared to either plate or nail fixation (*p* = 0.59).

## Discussion

This study is the first to directly compare native and periprosthetic distal femur fractures. PDFF were found to be commonly isolated injuries with complete metaphyseal comminution, affecting elderly women and those with low bone quality. NDFF, on the other hand, tended to occur in younger patients with less metaphyseal comminution and additional fractures. NDFF had increased revision reoperation rates compared to PDFF, specifically for I&D of the femur fracture of interest. In addition to having generally more open fractures in the NDFF group, patients with open fractures within the NDFF group were more likely to result in infection requiring I&D than those with closed fractures. Multivariate regression analysis revealed that NDFF were an independent risk factor for reoperation, specifically I&D of the fracture of interest, compared to PDFF.

The present study reports a PDFF gender distribution similar to Elsoe et al., who documented a sample of mostly females with a mean age of 77 years old [8]. Generally, our PDFF sample was characterized by a high prevalence of low bone density. Additionally, the PDFF cohort had a higher rate of low bone density compared to the NDFF cohort, which can be explained by the higher average age and the female majority in the PDFF group [20]. Low bone density has been highlighted as a risk factor for femur fractures in past studies, and low-energy distal femur fractures are now considered fragility fractures [6, 21]. Although trauma mechanisms were not formally investigated in our study, we observed that PDFF were mostly isolated injuries, which is more suggestive of a low-energy trauma mechanism as proposed by prior studies [7, 22].

The most common fracture pattern for PDFF was extraarticular with complete metaphyseal comminution (AO/OTA 33 A.3). The increased metaphyseal comminution is likely related to both the presence of low bone quality and the TKA implant affecting the stress concentration locations of the fracture. Low bone quality leads to an overall decreased tolerance for withstanding forces. Additionally, with the presence of a TKA, the fracture cannot propagate into the joint, and more energy may be imparted to the metaphysis.

While previous studies have shown that rIMN and lateral locked plating are the most common methods for treating PDFF, our study revealed that rIMN and NPC fixation are the most common methods utilized at our institution [10, 23, 24]. Further analysis of NPC fixation rates revealed an increase in prevalence from 3.3% in 2015 to 13.3% of all fixation constructs used for PDFF in 2019. There was no significant difference in malunion/nonunion rates for those treated with NPC fixation compared to those treated with rIMN or plate fixation alone for either NDFF or PDFF. Regarding postoperative weightbearing status, there was no significant difference in WBAT assignment for NDFF NPC fixation compared to nail or plate fixation (*p* = 0.88). This was likely confounded by the high prevalence of polytrauma and concomitant fractures in this group, which would have limited weightbearing. However, PDFF treated with NPC fixation were significantly more likely to be WBAT compared to those treated with nail or plate fixation alone (*p* < 0.05). NPC fixation is a recently being used as an ideal treatment for osteoporotic distal femur fractures (both PDFF and NDFF) due to the balanced energy distribution between bone and implant [25]. Currently, only small cohort studies exist which have found no significant difference in nonunion rates between NPC compared to nail or plate fixation, although a multicenter propensity analysis suggested there may be significantly lower nonunion rates in DFF treated with NPC fixation [25–27]. In addition to potentially reducing the risk of nonunion, many surgeons see a biomechanical advantage of combination fixation to facilitate early weightbearing in elderly patients [25]. It is of the authors’ opinion that the results reflect an increasing preference for this treatment by orthopedic surgeons at our institution to stabilize fractures in elderly patients with low bone density to facilitate earlier mobilization/weight bearing.

We have identified a large NDFF population of middle-aged patients (average age = 57 years old) with a balanced sex distribution. The most common fracture pattern consisted of metaphyseal comminution with intra-articular extension (AO/OTA 33 C.2), suggesting a predominantly high-energy trauma mechanism. This contrasts with the findings of Roy et al., who reported a small sample of NDFF at a level-1 trauma center (*n* = 87) consisting mostly of middle-aged female patients with comparable rates of high-energy (47%) and low-energy injuries (53%) [28]. These differences may be reflective of differences in the demographics of the catchment area that our institution serves.

In contrast to prior literature, which reports coronal plane (AO/OTA 33B.3) fractures representing 38% of all partial articular (AO/OTA 33B) native fractures, our study reports an overall rate of 14% for 33B fractures with a majority being fractures of the lateral condyle (AO/OTA 33B.1) [29]. The mechanism of coronal plane fractures consists of vertical shear forces experienced during high-energy trauma, which is consistent with our findings in the NDFF population. The difference in our reported prevalence of 33B.3 fractures is less likely to be explained by low detection as CT scans were obtained for all patients. These demographic and injury severity differences may be reflective of population differences in sampling; however, we report an NDFF cohort that is much larger than the previously mentioned study, with greater potential for generalizability.

Regarding the treatment of NDFF, rIMN was also the most common fixation method used, followed by lateral plating, which contrasts with previous studies which report plating as the most common fixation for NDFF [7, 11, 12]. Additionally, NPC was the third most common construct employed, which may reflect its increasing popularity as a treatment alternative for distal femur fractures as well as institutional preference for nailing.

Quality of reduction was improved in the PDFF cohort compared to the NDFF cohort, based on normative values of alignment. This may be due to the simplicity of the fractures as PDFF were all type A fractures whereas NDFF commonly had intra-articular components. Additionally, the TKA implants force a certain nail start point given the box component with less variation so perhaps the nail is more on axis. However, nailing of these fractures has been previously associated with malalignment [30, 31]. Finally, the increase in NPC versus lateral plating alone may account for some of the differences as the tendency for malreduction with a single lateral locked plate is well documented [32]. We did not observe any difference in nonunion, similar to prior studies but with an overall lower rate [30].

Contrary to our hypothesis, there were notable differences in outcomes between NDFF and PDFF. NDFF had significantly longer length of stays and were more likely to return to the operating room for additional treatment of the femur fracture compared to PDFF. The most common fracture pattern seen in NDFF undergoing reoperation was complete articular (AO/OTA 33 C), and the most common etiology for UROR was for irrigation and debridement of an initially open fracture due to infection risk, which is reflective of the more severe soft tissue injury and propensity for open fractures. Upfill-Brown et al. previously conducted a large retrospective review which found no significant differences in 30-day reoperation rates between PDFF and NDFF [13]. However, their study did not account for mechanism or fracture complexity. The differences in length of stay and UROR rates between the PDFF and NDFF groups noted in our study can be explained by the high prevalence of polytrauma, additional fractures, and increased fracture complexity (AO/OTA 33 C) in the NDFF group. Additionally, Kaufman et al. studied outcomes in a matched cohort of PDFF and NDFF and found that when controlling for age, sex, and injury severity, there was no significant difference in length of stay or < 90-day readmission rates between the two injuries [14]. The results of Kaufman et al. and our study support the notion that the risk for readmission is more closely tied to population-specific risk factors such as demographics, mechanism, and additional injuries than to the presence of periprosthetic fractures.

There are several notable strengths to this study. This investigation encompasses recent patient data from a large population spanning 8 years followed longitudinally. Our study takes into consideration the quality of reduction when assessing outcomes for PDFF and NDFF. Limitations to the study include retrospective-single site sampling and an unmatched patient cohort. Reduction quality measurements were made by a single grader, potentially introducing information bias. Our results reflect the treatment of distal femur fractures at a level-1 trauma center, and it is unclear whether similar results would be observed at a community hospital or arthroplasty surgical center. Finally, the addition of patient-reported outcomes would be beneficial but were not collected during the time frame investigated.

## Conclusion

In conclusion, there are differences in the patient demographics, fracture patterns, and fixation construct strategies of PDFF and NDFF patient populations. PDFF frequently occur as isolated, extra-articular, and comminuted injuries. Elderly women and those with poor bone quality are at high risk for PDFF. NDFF often occur in middle-aged individuals of both genders, often involving intra-articular extension and are frequently accompanied by additional orthopedic injuries. Patients with NDFF are at a significantly greater risk of reoperation, particularly due to soft tissue complications from open fractures. Finally, although rIMN was the common fixation strategy for both fracture categories, rates of NPC fixation for PDFF are increasing at our institution, likely to facilitate earlier weight bearing in elderly, nonpolytraumatized patients with qualifying fracture morphology. These differences can guide future research to enhance treatment algorithms and implant designs specific to each population, ultimately improving patient outcomes.

## Data Availability

No datasets were generated or analysed during the current study.
